# Knowledge and Attitude of Iranian Physicians towards Organ and Tissue Donation

**Published:** 2014-05-01

**Authors:** M. Salmani Nadoushan, B. Nozary Heshmati, A. Shabanzadeh Pirsaraee, I. Salmani Nodoushan, R. Jafari Nadoushan, F. Yazdi

**Affiliations:** 1 *Industrial Disease Research Center, Shahid SadoughiUniversity of Medical Sciences, Yazd, Iran*; 2 *Iranian Tissue Bank and Research Center, Tehran University of Medical Sciences, Tehran, Iran*; 3 *Department of Physiology and Electrophysiology Research Center, School of Medicine, Tehran University of Medical Sciences, Tehran, Iran *; 4 *Faculty of Health, Shahid Sadoughi University of MedicalSciences, Yazd, Iran*; 5 *Intensive Care Unit, Fayazbakhsh Hospital, Tehran, Iran*

**Keywords:** Attitude, Knowledge, Physicians, Organ donation, Organ transplantation, Tissue and organ procurement, Health personnel, Health knowledge, attitudes, practice

## Abstract

Background: Nowadays, organ transplantation is the treatment of choice for end-stage organ failure, which increases the importance of organ procurement. It seems that the attitude towards organ donation and transplantation affects people’s satisfaction. Moreover, health care personnel, especially physicians, should be familiar with transplantation rules and standards. It seems that understanding the knowledge and attitude of this group can affect the transplantation center policies.

Objective: To assess knowledge and attitude of a group of Iranian physicians towards organ and tissue donation.

Methods: In a cross-sectional study, 560 Iranian physicians including nephrologists, urologists and internists were asked to fill out a validated questionnaire containing their demographics, knowledge and attitude towards organ and tissue donation and transplantation.

Results: Of 560 participants, 435 (78%) agreed with organ donation after death and 285 (51%) agreed with living kidney donation. The most common reason provided by those who agreed with donation was “helping people” whereas the most common cause of disagreement was “to honor the body.” Only 32 (6%) physicians had no knowledge about transplantation rules. Complete awareness about theoretical basis and financial issues of transplantation was observed in 265 (47%) and 221 (40%) participants, respectively.

Conclusion: Physicians had a good attitude towards organ and tissue transplantation although less than half of them had knowledge of transplantation rules and its theoretical basis and financial issues; therefore, additional awareness and education of physicians is needed in all areas of the organ donation process in Iran.

## INTRODUCTION

Nowadays, organ transplantation is the treatment of choice for end-stage organ failure. There is discrepancy between organ demand and supply for organ transplantation, which can increase the importance of organ procurement. More than one million people in the world have received organ transplants, some of whom have survived for more than 25 years. The five-year survival rate is more than 70% in most cases. Although there are some treatments available for organ failure (*eg*, dialysis in renal failure), there are many patients on the waiting list for organ transplantation because of their problems. The main causes for refusal of organ donation are lack of knowledge and misinformation regarding organ donation [1]. Health care personnel, especially physicians, have an important role in shaping public attitudes, particularly when there is a considerable lack of knowledge about organ donation in the general population [2]. This role can be obtained by educating patients and encouraging families to discuss about organ and tissue donation [3]. Moreover, the physicians’ knowledge of organ donation rules and standards has positive effects on their attitudes [1], and thus, their role in shaping the general population’s attitudes. Assessment of the knowledge and attitudes of our physicians and medical staff towards transplantation can be used in future planning programs for increasing the rate of organ and tissue donation. In this study, we assessed knowledge and attitudes of nephrologists, urologists and internists towards organ and tissue donation.

## MATERIALS AND METHODS

This cross-sectional survey was conducted on 560 Iranian specialists including nephrologists, urologists and internists selected by random cluster sampling from Tehran municipality districts. The participants were given a self-administered questionnaire that included the following information:

1. Demographics including age, sex, marital status, specialty, and job situation

2. Attitudes towards organ and tissue donation including

Attitude towards cadaveric donation and the reasons

Attitude towards living kidney donation and the reasons

Attitude towards cadaveric donation of a family member

Attitude towards cadaveric donation of themselves

Attitude towards having donation card

Attitudes towards getting consent of organ and donation

Amount of reliance to medical staff in charge of transplantation

3. Knowledge about…

transplantation rules in Iran

theoretical basis of transplantation

financial issues of transplantation

## RESULTS

A total of 294 internists, 129 urologists, and 31 nephrologists with different levels of experience in organ donation answered the questionnaire. The participants included 366 men and 159 women. The mean±SD age of the participants was 47.7±18.2 (range: 24–74) years. Of the studied physicians, 412 (81%) were married and 96 (19%) were single; 435 (78%) agreed with organ donation after death. The most important reason providing for their agreement in this group was “to help others” whereas the most common reason for disagreement was “to honor the body.” Of studied physicians, 285 (51%) agreed and 123 (22%) completely disagreed with living kidney donation. The most common reason for agreement and disagreement on living kidney donation was “to help others” and “probable damages to donors,” respectively. Physicians with negative attitude towards living kidney donation were mostly opposed organ and tissue donation after death (p<0.05). When we asked the specialists about having a donation card, only 68 (12%) responded positively; however, 437 (78%) physicians expressed a positive attitude towards having a donation card (Table 1).

**Table 1:** The rate of donation card possession in physicians with different attitudes

Total	Donation card				
	No answer	No	Yes		
435	4	367	64	Positive	Attitudes towards organ and tissue donation after death
107	3	100	4	Negative	
18	2	16	0	No answer	
560	9	483	68		Total

Almost half of the studied physicians had a will to donate all their organs and tissues after death followed by the kidneys, liver, heart, cornea, lungs, pancreas, heart valves, skin, and bone. Moreover, 367 (66%) physicians agreed to give consent for organ donation whereas 40 (7%) completely disagreed. Only 32 (6%) physicians had no knowledge about transplantation rules. On the other hand, 265 (47%) physicians were aware of the theoretical basis of transplantation and 333 (60%) did not have enough information about its financial issues in Iran. There was a significant correlation between physicians’ attitudes toward organ and tissue donation after death and knowledge about transplantation rules (p<0.05) and financial issues of transplantation in Iran (p<0.05) (Table 2). 

**Table 2:** Correlation between physicians’ knowledge and their attitude towards organ and tissue donation after death

p value	Attitude towards organ and tissue donation after death			Knowledge about…	
	No answer	Negative	Positive		
<0.001	17	95	414	Yes	transplantation rules
	0	11	21	No	
	1	1	0	No answer	
0.042	5	35	182	Yes	financial issues of transplantation in Iran
	12	70	251	No	
	1	2	2	No answer	
0.385	8	51	206	Yes	theoretical basis of transplantation
	3	34	140	No	
	7	22	89	No answer	

There was a significant correlation between physicians’ knowledge and their attitude towards living kidney donation (p<0.05) (Table 3). 

**Table 3:** Correlation between physicians’ knowledge and their attitude towards living kidney donation

p value	Attitude towards living kidney donation				Knowledge about…	
	No answer	No opinion	Negative	Positive		
<0.001	4	164	93	265	Yes	transplantation rules
	0	11	4	17	No	
	1	0	1	0	No answer	
<0.001	2	62	32	126	Yes	financial issues of transplantation in Iran
	2	110	65	156	No	
	1	3	1	0	No answer	
0.001	1	79	57	128	Yes	theoretical basics of transplantation
	1	71	19	86	No	
	3	25	22	68	No answer	

Only 7 (1.5%) participants had no confidence in medical staff in charge of transplantation (6 were internists); however, there was no significant correlation between specialty and having confidence in the staff.

## DISCUSSION

We found that most of the specialists (78%) agreed with organ and tissue donation after death for the main reason of “helping others.” This finding was relatively similar to the results of studies conducted by Rios, *et* *al*, on future specialists in Spain and Mexico (93%) [4] and Amaral, *et al*, in Brazil (87%) [5]. When we considered the factors affecting the attitude towards organ and tissue donation after death [4, 6-8], there were two groups of variables among specialists: 1) emotional factors (according to their opinions) and 2) knowledge about and attitude towards organ and tissue donation and transplantation. Similar to Rios study [4], we found that one of the main factors affecting the attitude towards living kidney donation was knowledge and attitude towards organ donation and transplantation. We found that only 7% of the physicians were not willing to obtain consent for organ donation while in a study conducted in India, more than 25% of physicians and nurses felt that organ donation was not part of their professional study and were not willing to obtain consent [9]. We also observed specialists’ confidence in the medical staff in charge of transplantation, indicating the proper function of this team. 

We noted that of 78% of the physicians who believed in the concept of organ donation, only 12% had organ and tissue donation cards at the time of completing the questionnaire. Erdogan, *et al,* found that of 98% of the Turkish physicians with positive attitude towards organ donation, only 23% had organ donation cards. This difference may have resulted from more motivational programs about organ and tissue donation and transplantation and more availability of donation cards for physicians in Turkey as compared to Iran [1].

In our study, 66% of the physicians had inclination for getting consent, so it will be useful for organ transplantation teams to get consent before they come to make patients with brain death ready for recovery of organs more rapidly and easier, hence, more organ suitability. Although we expected specialists to have more knowledge about transplantation, we found that we should provide suitable conditions to improve the knowledge of physicians and other people. Therefore, it is in the best interest of transplantation centers all over the world to devise programs to increase the knowledge and positive attitude of the general population towards organ donation to increase life expectancy in needy patients and enhance public health.
